# Comparing Sensitivity and Specificity of Ultrasonography With Chest Radiography in Detecting Pneumothorax and Hemothorax in Chest Trauma Patients: A Cross-Sectional Diagnostic Study

**DOI:** 10.7759/cureus.44456

**Published:** 2023-08-31

**Authors:** Aswin K, Balamurugan S, Ramkumar Govindarajalou, Ganesh Kumar Saya, Elamurugan TP, Gunaseelan Rajendran

**Affiliations:** 1 Emergency Medicine, Indira Gandhi Medical College and Research Institute, Puducherry, IND; 2 General Surgery, Jawaharlal Institute of Postgraduate Medical Education and Research, Puducherry, IND; 3 Radiodiagnosis, Jawaharlal Institute of Postgraduate Medical Education and Research, Puducherry, IND; 4 Preventive and Social Medicine, Jawaharlal Institute of Postgraduate Medical Education and Research, Puducherry, IND; 5 Emergency Medicine, Aarupadai Veedu Medical College and Hospital, Vinayaka Mission's Research Foundation, Puducherry, IND

**Keywords:** chest radiography, pocus, specificity, sensitivity, hemothorax, pneumothorax, chest trauma

## Abstract

Background

Thoracic trauma accounts for 20-25% of all traumas and is the third most frequent cause of death, after abdominal injury and head trauma. In the Emergency Department (ED), shifting an unstable patient to the X-ray room for detecting pneumothorax and hemothorax is always risky and bedside X-ray causes radiation exposure not only to the particular patient but also to the surrounding patients in a congested and busy ED. This can be avoided by using bedside ultrasonography (USG) as the initial imaging modality in chest trauma patients.

Objective

To compare the sensitivity and specificity of ultrasonography and chest radiography in detecting pneumothorax and hemothorax in chest trauma patients.

Methods

This cross-sectional diagnostic study was conducted for a period of one year at Jawaharlal Institute of Postgraduate Medical Education and Research, Puducherry, India, a tertiary care centre. All consecutive patients (n=255) with a suspected history of chest trauma were included in the study. The patients were evaluated bedside using USG by point of care ultrasonography trained emergency medicine physician and subsequently underwent chest radiography for documentation of pneumothorax and hemothorax. Sensitivity and specificity were calculated for ultrasonography and chest X-ray (CXR) compared with the composite gold standard (chest radiography and computed tomography thorax).

Results

Of the 255 patients, 89% were males. The mean age of the patients was 43.46 (standard deviation 16.3). Road traffic accident (RTA) was the most common mode of injury (81%). The median (interquartile range) time interval between injury and arrival at the hospital was four hours (2.5-7). About 16.1% of the patients had subcutaneous emphysema. About 88.2% of the patients were hemodynamically stable and 78% of the patients had associated other system injuries. The sensitivity and specificity of USG in detecting pneumothorax were 85.7% and 95.3% respectively and that of CXR were 71.4% and 100% respectively. Our study found that the sensitivity and specificity of USG in detecting hemothorax were 79% and 97.9% respectively and that of CXR were 62.9% and 100% respectively. Even in the subset of patients in whom a computed tomography scan was done, the sensitivity of USG was higher than that of CXR in detecting pneumothorax and hemothorax. The specificity of USG in detecting pneumothorax was the same as that of CXR and the specificity of USG in detecting hemothorax was higher than that of CXR in that subset of patients.

Conclusion

The sensitivities of USG in detecting pneumothorax and hemothorax were higher than that of CXR. The specificities of USG in detecting pneumothorax and hemothorax were comparable to that of CXR. Hence bedside USG performed by emergency physician during resuscitation helps in rapid diagnosis and early management of chest trauma patients.

## Introduction

With rising population, accelerated urbanization, industrialization, and drastic increase in vehicular transport, trauma still continues to be a serious threat to global public health as it is linked to high rates of morbidity and mortality in both developed and developing nations, with an estimated six million deaths worldwide [1]. In patients with polytrauma, thoracic trauma accounts for 20-25% of all traumas and is the third most frequent cause of death, behind abdominal injury and head trauma [2,3]. Blunt chest injuries are more common than penetrating injuries and can potentially pose a threat to the airway, breathing, and circulation in the traumatized patient, thus directly affecting the clinical course and outcome.

Imaging studies play an important role in the workup of the patient with thoracic trauma. The chest radiograph is the initial imaging study usually done in most of the trauma centers in India as it is quick, easy to get, cost-effective, and has less radiation exposure to the patients. However, a bedside chest X-ray (CXR) has very low sensitivity and most of the injuries can be missed. Hence computed tomography (CT) has to be used in the evaluation of chest trauma. Recent studies have shown that ultrasonography (USG) done in the emergency room has been found to be useful in the initiation of early resuscitation and management [4-8]. Though some studies have shown that ultrasonography has better sensitivity and specificity in detecting pneumothorax and hemothorax in chest trauma patients, a study published in 2016 has concluded that USG is not reliable in the detection of injuries without bleeding or free fluid and is highly operator-dependent [4]. In most of the published studies USG has been done by the radiologist whereas in the current study the evaluation was done by the resuscitating emergency physician. Also very few Indian studies are there comparing the sensitivity and specificity of USG and CXR in chest trauma patients. Therefore, the purpose of this study is to compare the sensitivity and specificity of ultrasonography and chest radiography in detecting pneumothorax and hemothorax in chest trauma patients.

## Materials and methods

This cross-sectional diagnostic study was conducted for a period of one year at Jawaharlal Institute of Postgraduate Medical Education and Research a tertiary care hospital in south India for a period of one year after obtaining approval from the Jawaharlal Institute of Postgraduate Medical Education and Research (JIPMER) Institutional Ethics Committee (JIP/IEC/2016/1030). All consecutive patients with suspected chest trauma attending the Department of Emergency Medicine and Trauma were included in the study. Patients with a history of penetrating chest trauma and pregnant females were excluded from the study. The estimated sample size (n= 255) was calculated using OpenEpi v 3 (Open Source Epidemiologic Statistics for Public Health Version 3, Atlanta, GA) with a 5% level of significance, 80% power and using the sensitivity (83.6%) of ultrasonography in detecting pneumothorax as reported by Vafaei et al. [4].

The study population was evaluated bedside using ultrasonography by a Point of Care Ultrasonography (POCUS) trained emergency medicine physician. Diagnosis of pneumothorax was done by examining the intercostal space (second-sixth) bilaterally in the supine position, by using a high-frequency linear probe. The pleural line was identified and examined for four to five respiratory cycles for lung sliding, and the absence of lung sliding was taken as pneumothorax. For hemothorax, the lateral chest area above the diaphragm was examined in coronal view using a probe bilaterally. Visualization of anechoic areas in the pleural space was taken as hemothorax [9]. Subsequently, the patient underwent chest radiography for documentation of pneumothorax and hemothorax. Patients with strong clinical suspicion of pneumothorax/hemothorax but were negative for pneumothorax/hemothorax in chest radiography underwent a CT scan. Chest radiography and CT scan were interpreted by a radiologist who did not know the ultrasonography findings. The patient underwent further procedures/treatment as per the treating physician/surgeon.

Sensitivity and specificity were calculated for ultrasonography and chest radiography comparing with the composite gold standard (CT thorax or chest radiography). Data were collected using a structured proforma at the time of admission and when the patient underwent ultrasonography, chest radiography, and CT scan. Data were entered in Microsoft Excel (Microsoft Corporation, Redmond, USA) and analyzed. Continuous variables were expressed as mean and standard deviation (SD) or median and interquartile range (IQR) and categorical variables were expressed as proportions and percentages.

## Results

A total of 255 chest trauma patients were included in the study during the study period. There was a significant male preponderance (n=226) in the study population (p<0.005). The mean age of the patients was 43.46 years (standard deviation {SD} 16.3). Most of the patients were daily wage laborers (70%) and road traffic accident (RTA) was the most common mode of injury (81%). The median (IQR) time interval between injury and arrival at the hospital was four hours (2.5-7). Chest wall tenderness was seen in 81.2% of the patients, 36.9% of patients had bony crepitus, 20.4% had chest wall contusion, and subcutaneous emphysema was present in 16.1%. Among 255 patients, 198 patients had at least one injury other than the chest injury like head injury, abdominal injury, pelvis injury, or long bone injury and 88.2% were hemodynamically stable. About 73.7% had a Glasgow Coma Scale (GCS) between 13 and 15 and 20.4% had a GCS less than nine. 

Using ultrasonography, 63 (24.7%) and 53 (20.8%) patients were diagnosed to have pneumothorax and hemothorax respectively out of the total 255 patients. Of 63 patients who were diagnosed to have pneumothorax, 61 had unilateral pneumothorax, and two had bilateral pneumothorax and of the 53 patients who were diagnosed to have hemothorax, 51 had unilateral hemothorax and two had bilateral hemothorax.

Using CXR, 45 (17.6%) and 39 (15.3%) patients were diagnosed to have pneumothorax and hemothorax respectively out of the 255 patients. Of 45 patients who were diagnosed to have pneumothorax, 44 had unilateral pneumothorax, one had bilateral pneumothorax, and of the 39 patients who were diagnosed to have hemothorax, all had unilateral hemothorax. Tables 1-2 show the sensitivity and specificity of detecting pneumothorax and hemothorax with composite gold standard and with CT scan respectively.

**Table 1:** Sensitivity and specificity of detecting pneumothorax and hemothorax when the composite gold standard was considered as the gold standard (n=255)

	Pneumothorax		Hemothorax	
	Ultrasonography (%)	Chest X-ray (%)	Ultrasonography (%)	Chest X-ray (%)
Sensitivity	85.7	71.4	79	62.9
Specificity	95.3	100	97.9	100

**Table 2:** Sensitivity and specificity of detecting pneumothorax and hemothorax when CT scan was considered as the gold standard (n=60)

	Pneumothorax		Hemothorax	
	Ultrasonography (%)	Chest X-ray (%)	Ultrasonography (%)	Chest X-ray (%)
Sensitivity	81.8	45.5	65.7	34.3
Specificity	92.6	92.6	96	92

## Discussion

In the Emergency Department (ED), shifting an unstable patient to the X-ray room is always risky and bedside X-ray causes radiation exposure not only to the particular patient but also to the surrounding patients in a congested and busy ED. This can be avoided by using bedside USG as the initial imaging modality in chest trauma patients. In our study, 89% of the subjects were males and 11% were females. This is similar to other studies done in India and other countries [10-12]. This pattern of male preponderance can be explained by the fact that mostly males are involved in outdoor activities. Most of the subjects were of the age group 21-40 years (41.6% in our study). This is similar to other studies because the breadwinner of the family will be of this age group and will be more involved in travelling [13,14].

In the present study, we found that the most common clinical presentation was chest tenderness which was present in 81.2% of our patients which is similar to other studies [12,15]. We observed that associated injuries were present in 78% of our patients along with chest injury in our study. A similar proportion was found in another Indian study done by Iyer et al. [11]. The most common associated injury is head injury in our study which comprised around one-third of the patients. A similar high incidence of head injury was found in another Indian study done by Choudhary et al. [10]. However, in another study done outside India by Horst et al., the most common associated injury was of the extremities (one-fourth of the patients) followed by head injury (one-fifth of the patients) [16]. This is because helmets and seat belts are not commonly used while travelling in two wheelers in our region increasing the incidence of head injuries.

In our study, the sensitivity and specificity of USG in detecting pneumothorax were 85.7% and 95.3% respectively. This is consistent with the study done by Vafaei et al., who found that the sensitivity and specificity of USG in detecting pneumothorax were 83.6% and 97.9% respectively [4]. A meta-analysis done in 2014 by Ebrahimi et al., showed that the pooled sensitivity and specificity were 87% and 99% respectively [7]. A study done in 2016-2017 by Salama et al., detected the sensitivity and specificity to be 81% and 100% respectively for pneumothorax [12]. The USG window was not clear in 41 patients who had subcutaneous emphysema in our study. This could have reduced the sensitivity and specificity of our study to some extent.

The sensitivity and specificity of CXR in detecting pneumothorax in our study were 71.4% and 100% respectively. Vafaei et al. observed that they were 67.3% and 92.7% respectively [4]. The meta-analysis by Ebrahimi et al. detected them as 46% and 100% respectively [7]. Salama et al’s study showed the sensitivity and specificity as 75% and 88.9% respectively [12]. The higher specificity of CXR in our study was due to the reason that in our study, the gold standard was taken as CXR in patients who had clinical suspicion of chest injury and positive findings in CXR. CT scan was considered as the gold standard only in the patients who had a strong suspicion of chest injury but negative findings in CXR. This was because we were not able to do CT scans for all patients as it is not the protocol in our institution. Only patients who had strong clinical suspicion but negative findings in CXR underwent CT scans. However, since CXR has good specificity according to most of the studies, this limitation is acceptable [7,17].

Our present study found that the sensitivity and specificity of USG in detecting hemothorax were 79% and 97.9% respectively. This finding was consistent with most of the other studies. Vafaei et al. observed that the sensitivity and specificity were 75.9% and 95.9% respectively [4]. A meta-analysis by Movaghar et al. detected the pooled sensitivity and specificity as 67% and 99% [17]. The sensitivity in detecting hemothorax is poorer than in detecting pneumothorax in our study which is comparable to the study done by Vafaei et al. [4]. In patients who had concomitant pneumothorax with massive subcutaneous emphysema, detection of hemothorax was found to be difficult. In those patients, careful USG examination was needed.

The sensitivity and specificity of detecting hemothorax by CXR in our study were found to be 62.9% and 100%, respectively. The study by Vafaei et al. showed that the sensitivity and specificity were 58.6% and 95.1%, respectively [4]. A meta-analysis by Movaghar et al. showed that the pooled sensitivity and specificity were 54% and 99% [17]. The higher specificity of CXR is explained by the fact that CXR was considered the gold standard for a significant number of patients as described earlier. The sensitivity of USG in detecting pneumothorax and hemothorax was higher than that of CXR. The specificity of USG in detecting pneumothorax and hemothorax was less than that of CXR when the composite gold standard was considered. However, the specificity of USG was comparable to that of CXR.

Strengths and limitations

The limitation of using a composite gold standard in our study is acceptable because in the meta-analysis done by Ebrahimi et al., in detecting pneumothorax and in the meta-analysis done by Movaghar et al., in detecting hemothorax, the pooled specificities of CXR were 100% and 99% respectively which are significantly higher values [7,17]. Hence CXR was taken as the gold standard in patients with clinical suspicion and positive CXR findings. In our study, subcutaneous emphysema made it difficult to interpret a few USG findings and the composite gold standard was only used as the gold standard. In spite of these limitations, we observed that USG had a better sensitivity compared to CXR.

Even in the subset of patients in whom a CT scan was done, the sensitivity of USG was higher than that of CXR in detecting pneumothorax and hemothorax. The specificity of USG in detecting pneumothorax was the same as that of CXR, and the specificity of USG in detecting hemothorax was higher than that of CXR in that subset of patients. An additional advantage of our study is that it was done by an emergency physician who knew about the clinical features of the patients in detail that had led to a better pretest probability. Also, the time delay for patient shifting to the radiologist room for USG was avoided.

## Conclusions

The sensitivities of USG in detecting pneumothorax and hemothorax were higher than that of CXR in chest trauma patients. The specificities of USG in detecting pneumothorax and hemothorax were less than that of CXR when the composite gold standard was considered, but the differences were marginal. However, the specificities of USG in detecting pneumothorax and hemothorax were the same and higher respectively as compared to CXR when CT scan was considered as the gold standard.

From our study, we conclude that bedside USG performed by emergency physicians during resuscitation is as good as CXR in detecting pneumothorax and hemothorax in chest trauma patients due to higher pretest probability. It further helps in rapid diagnosis and early management of life-threatening chest injuries. However, more Indian studies are required since our study is a single-centre study.
